# BNT162b2 mRNA COVID-19 Vaccine Effectiveness in the Prevention of SARS-CoV-2 Infection and Symptomatic Disease in Five-Month Follow-Up: A Retrospective Cohort Study

**DOI:** 10.3390/vaccines9101143

**Published:** 2021-10-07

**Authors:** Francesco Paolo Bianchi, Silvio Tafuri, Giovanni Migliore, Luigi Vimercati, Andrea Martinelli, Annamaria Lobifaro, Giusy Diella, Pasquale Stefanizzi, on behalf of the Control Room Working Group

**Affiliations:** 1 Department of Biomedical Science and Human Oncology, Aldo Moro University of Bari, Piazza Giulio Cesare 11, 70124 Bari, Italy; francesco.bianchi@asl.brindisi.it (F.P.B.); andrea.martinelli@uniba.it (A.M.); annamarialobifaro@gmail.com (A.L.); giusy.diella@uniba.it (G.D.); pasquale.stefanizzi@uniba.it (P.S.); 2 Hospital Director, Bari Policlinico General Hospital, 70124 Bari, Italy; giovanni.migliore@policlinico.ba.it; 3 Department of Interdisciplinary Medicine, Aldo Moro University of Bari, 70124 Bari, Italy; luigi.vimercati@uniba.it

**Keywords:** COVID-19, documented infection, vaccine effectiveness, healthcare workers

## Abstract

To combat the COVID-19 pandemic, a mass vaccination campaign was initiated in Italy on 27 December 2020. The vaccine available to immunize Italian healthcare workers (HCWs) was the BNT162b2 mRNA COVID-19 vaccine (Comirnaty). This study evaluated the effectiveness of the vaccine against documented SARS-CoV-2 infection and symptomatic diseases in the medium- to long-term. HCWs at Bari Policlinico University-Hospital (Italy) who completed the vaccination schedule were matched with HCWs who had refused vaccination; the two groups were followed-up for 5 months (January–May 2021). Vaccine effectiveness (VE) against infection was 97.7% (95.4–99.0%) at 14–34 days after the first dose, and 94.8% (87.0–97.8%), 83.0% (65.0–92.0%), and 81.0% (42.0–94.0%) at 14–41, 42–69, and >69 days, respectively, after the second dose. The estimated VE for documented symptomatic disease was 99.2% (96.4–99.8%) at 14–34 days after the first dose and 97.2% (90.3–99.2%), 85.0% (63.0–94.2%), and 88.0% (42.0–97.6%) at 14–41, 42–69, and >69 days, respectively, after the second dose. Efforts to increase vaccination rates should be strengthened, including mandatory vaccination for HCWs and greater incentives to increase vaccine acceptance by the general population.

## 1. Introduction

COVID-19, the infectious disease caused by the novel coronavirus SARS-CoV-2, has reached global proportions and has been considered a pandemic since mid-2020 [1]. According to the World Health Organization (WHO), there have been ~231,000,000 confirmed cases of COVID-19 globally, including more than 4,700,000 deaths (data until 28 September 2021) [2]. The European Centre for Disease Prevention and Control has reported >37,000,000 cases and >750,000 deaths in the EU/EEA (data until 23 September 2021) [3]. In Italy, over 4,600,000 cases have been documented, including 143,000 cases in healthcare workers (HCWs) and 130,000 COVID-19-related deaths (fatality rate: 2.8%) [4].

To combat the COVID-19 pandemic, a mass vaccination campaign was initiated in Italy and other European countries on 27 December 2020. The Italian government opted to prioritize the vaccination of HCWs, a decision in line with the recommendations of the Centers for Disease Control and Prevention (CDC) [5]. As they provide critical care to patients who are or might be infected with SARS-CoV-2, HCWs are at high risk of exposure to the virus and thus to the development of COVID-19; furthermore, vaccinating HCWs safeguards healthcare capacity. The vaccine available to immunize HCWs was the BNT162b2 mRNA COVID-19 vaccine (Comirnaty, from Pfizer and BioNTech, Mainz, Germany), the first vaccine to be approved by the European Medicines Agency. It is indicated for individuals 12 years of age and older and is administered in two doses delivered at least 21 days apart [6,7].

In the pre-licensure trial, the vaccine had a 95% efficacy in preventing COVID-19, including severe disease [8]. Aside from transient local and systemic reactions, no safety issues were identified [8]; however, no information was released on the vaccine’s efficacy against documented Sars-CoV-2 infection.

In 2021, an observational study [9] investigated the effectiveness of the BNT162b2 mRNA vaccine in >1,000,000 Israeli inhabitants (half fully vaccinated and half unvaccinated) during the period from 20 December 2020 to 1 February 2021. The vaccine was shown to prevent symptomatic illness, with an effectiveness of 94% 7 days after the second dose. The same study investigated vaccine effectiveness (VE) in preventing documented infection. The results showed that the estimated VE against documented infection was 46% (95% confidence interval [CI]: 40–51%) at 14–20 days after the first dose, 60% (95%CI: 53–66%) at 21–27 days after the first dose, and 92% (95%CI: 88–95%) in the follow-up period starting 7 days after the second dose.

A brief report of our research team, published in May 2021, evaluated VE in a sample of HCWs followed-up from 27 December, 2020 to 31 January, 2021. The VE for documented infection was 61.9% (95%CI: 19.2–82.0%) at 14–20 days after the first dose, 87.9% (95%CI: 51.7–97.0%) at 21–27 days after the first dose, and 96.0% (95%CI: 82.2–99.1) at ≥7 days after the second dose [10].

In this study, we evaluated the medium- to long-term effectiveness of the BNT162b2 mRNA vaccine against SARS-CoV-2 infection and symptomatic disease in all of the immunized HCWs of Bari Policlinico General Hospital. The results obtained for this large population were compared with those of our previous report [10]. The study was carried out in Apulia (southern Italy, ~4,000,000 inhabitants), where, from February 2020 to 7 July 2021, 253,571 confirmed cases of COVID-19 and 6646 related deaths were reported [11].

## 2. Materials and Methods

Our observational cohort study was conducted at Bari Policlinico General University-Hospital (1000 beds, 6000 HCWs), where 180 hospital beds are reserved for COVID-19 patients. The vaccination campaign for HCWs was started on 27 December 2020, with scheduling and follow-up activities coordinated by the Hygiene and Occupational Medicine departments of Bari Policlinico.

All vaccinations are administered by public health physicians who are experts in vaccinology. The two doses of BNT162b2 mRNA vaccine are delivered intramuscularly in the deltoid muscle at least 21 days apart. Thus far (April 2021), vaccination prophylaxis is not mandatory and HCWs can refuse vaccination. Informed consent is collected at the time of vaccination. All vaccinated HCWs are followed-up for 1 month to assess the development of adverse effects.

Policlinico Bari General Hospital has also adopted a specific procedure for the control and prevention of SARS-CoV-2 infection. Asymptomatic HCWs are compulsorily screened every 14 days for SARS-CoV-2 infection using molecular tests on naso-pharyngeal swabs, obtained as recommended by the WHO [12]. Fast-track access to molecular testing is ensured for HCWs with signs and symptoms of COVID-19 (fever, cough, ageusia, etc.). A commercial real-time PCR assay (Allplex2019-nCoV Assay; Seegene, Seoul, Korea) is used to identify the presence of the E gene, RdRP gene, and N gene of SARS-CoV-2. Data on infection control and prevention are entered into the computerized GIAVA COVID-19 platform, as described below.

The population in this study comprised HCWs who had completed the basal vaccination routine (both doses) between 27 December 2020 and 31 March 2021. They were paired with Bari Policlinico HCWs who, during the same period, remained unvaccinated because they refused vaccination (with the exception of those infected during the first days of the vaccination campaign); the two groups were followed-up until 31 May 2021. HCWs with a documented history of SARS-CoV-2 infection before enrollment were excluded from participation in the study. Overall, 6136 HCWs were enrolled.

The overall vaccination status of HCWs was assessed using the Regional Immunization Database (GIAVA) [13].

Data on documented cases of SARS-CoV-2 infection were extracted from the surveillance platform GIAVA COVID-19, developed on the basis of the WHO Go. Data outbreak investigation tool [13] and set up to manage the pandemic emergency in Apulia. We considered all subjects for which a confirmed diagnosis of SARS-COV-2 infection from 1 January 2021 to 31 May 2021.

The final dataset was created as an Excel spreadsheet that included information on sex, age at enrollment, group (vaccinated vs. unvaccinated), documented infection (YES/NO), symptomatic disease (YES/NO), and duration of symptoms. An anonymized data analysis was performed using the STATA MP17 software. Continuous variables are reported as the mean ± standard deviation and range or median and interquartile (IQR) rante, and categorical variables as proportions. Skewness and kurtosis tests were used to evaluate the distribution of continuous variables; non-normally distributed variables were normalized using a normalization model. Student’s t-test for independent data was used to compare continuous variables between groups, and a chi-squared test was used to compare proportions.

The outcome of interest was documented SARS-CoV-2 infection as confirmed by a positive PCR test and COVID-19, defined as a SARS-COV-2 infection with the development of typical symptoms (fever, cough, etc.); any reinfection was recorded. Survival curves for the vaccinated and unvaccinated groups were plotted using the Kaplan–Meier estimator. A log-rank test was used to compare the two groups. Incidence rates per 1000 person days of infection and of disease were estimated, including 95% confidence intervals (95%CIs). The incidence rate ratio (IRR) and its 95%CI were also calculated. Four periods were considered: 14–34 days after the first vaccine dose, 14–-41 days after the second vaccine dose, 42–69 days after the second vaccine dose, and >69 days after the second vaccine dose until the end of follow-up. For each period, a risk ratio (RR) for vaccination vs. no vaccination was calculated. Vaccine effectiveness (VE), defined as one minus the RR, and its 95%CI were estimated. For all tests, a two-sided *p*-value <0.05 was considered to indicate statistical significance.

## 3. Results

The study population comprised 6136 HCWs: 5351 (87.2%) in the vaccinated group and 787 (12.8%) in the unvaccinated group. The characteristics of the participants at enrollment are presented in Table 1. The median time between the first and second doses was 23 days (IQR range = 22–23).

During a median follow-up period of 139 days (IQR = 135–143), there were 227 cases of SARS-CoV-2 infection (3.7%), of which 179/787 (22.7%) occurred in the unvaccinated group and 48/5.351 (0.9%) in the vaccinated group (*p* < 0.0001). The cumulative incidence was 2.8 per 10,000 person days: 19.9 per 10,000 person days in the unvaccinated group and 0.7 per 10,000 person days in the vaccinated group. The IRR was 0.03 (95%CI: 0.02–0.05; *p* < 0.0001).

Symptomatic disease developed in 131 (2.1%) HCWs, including 112 (14.3%) in the unvaccinated group and 19 (0.4%) in the vaccinated group (*p* < 0.0001). The cumulative incidence was 1.6 per 10,000 person days: 12.4 per 10,000 person days in the unvaccinated group and 0.3 per 10,000 person days in the vaccinated group. In the vaccinated group, the IRR was 0.02 (95%CI: 0.01–0.04; *p* < 0.0001). The recorded symptoms and their frequencies are described in Table 2.

**Figure 1:**
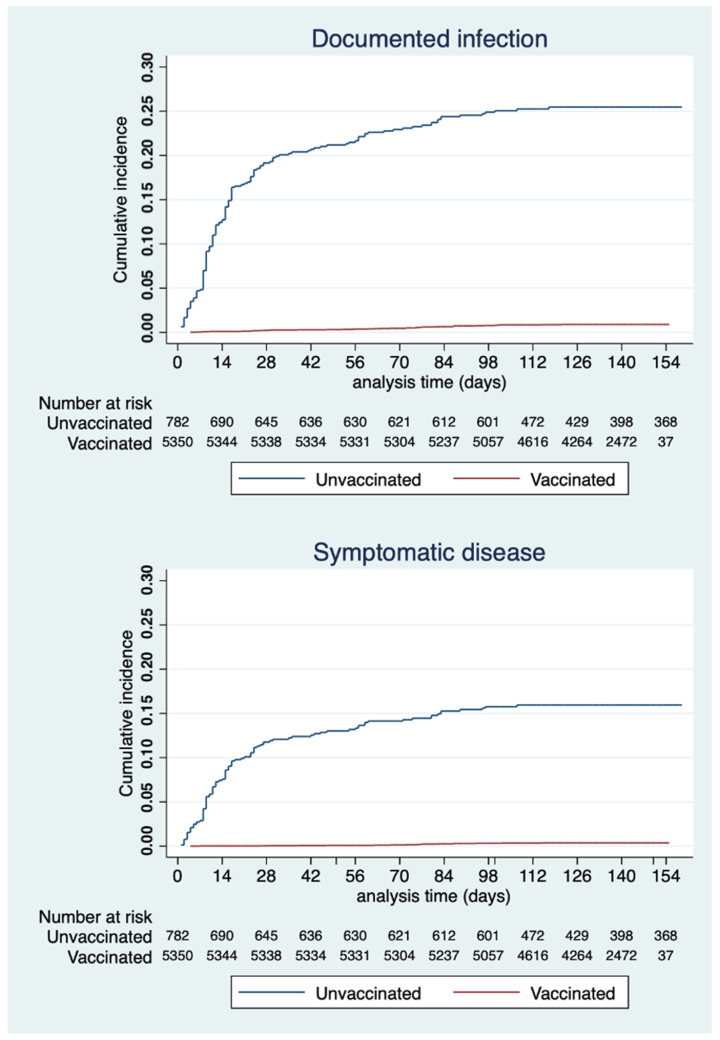
Cumulative incidence of documented infection and symptomatic disease, by group (vaccinated vs. unvaccinated), expressed using Kaplan–Meier estimator.

The cumulative incidence of both infection and symptomatic disease in the two groups (log-rank *p*-value < 0.0001 for both estimation) is shown in Figure 1.

The estimated VE values for documented infection and symptomatic disease are reported in Table 3.

Nine hospitalizations were reported, including eight (1.0%) HCWs in the unvaccinated group and one (0.02%) HCW in the vaccinated group (*p* < 0.0001).

During vaccination follow-up, there were no serious and/or long-term adverse reactions. The safety of the vaccine is the subject of a report currently in preparation.

## 4. Discussion

Our study showed that the effectiveness of the BNT162b2 mRNA vaccine for both documented infection and symptomatic disease decreased in the weeks following vaccine administration; nonetheless, at least under the conditions of this study, the vaccine is highly effective. The VEs determined in our study are slightly higher than those reported by Dagan N et al. [9], although the values at ≥7 days after the second dose are very similar (92% for symptomatic disease). However, the sample size of the two studies was very different and our study population consisted solely of HCWs, a group largely excluded by the other study. HCWs are clearly at higher risk of exposure to Sars-CoV-2, which would explain the larger number of infections in our unvaccinated group. Furthermore, since even vaccinated HCWs are periodically screened with a PCR test, to reduce the risk of nosocomial outbreaks, the results for this group are highly reliable and the risk of under-reporting was very low. In a study by Keehner J et al. [14], in a sample of 36,659 HCWs from the USA, 8 tested positive 8–14 days after the second vaccination, and 7 tested positive ≥15 days after the second vaccination; VE values were not reported. A study by Benenson S et al. [15], conducted in an Israeli hospital setting, showed that vaccination of HCWs with the BNT162b2 vaccine resulted in a major reduction in new cases of COVID-19 among those who had received both doses (incidence of COVID-19 x1,000 HCWs: 19.9 in unvaccinated vs. 1.0 in vaccinated). Finally, in a study by the CDC that examined the effectiveness of the Pfizer-BioNTech and Moderna mRNA vaccines in preventing SARS-CoV-2 infections among 3950 HCWs in the USA, the risk of infection was reduced by 90% at ≥2 weeks after the second dose [16]. Compared to our brief, previously published report [10], the VE values of this study are higher (88% vs. 95%), perhaps due to its larger sample size and the inclusion of HCWs not directly involved in patient care. Moreover, VE was determined after only a few weeks, not over the medium- to long-term.

The HCWs in our unvaccinated group were slightly younger than those in the vaccinated group and the vaccination rate was higher among physicians than among other healthcare professionals. In a previous report [17], however, older age was identified as a determinant of vaccination hesitancy/refusal, while being a physician was a determinant of vaccination compliance.

The strengths of our study are its very large sample size and a follow-up period longer than that of similar studies in the literature. The most important limitation was our inability to stratify VE according to sex, age, etc., due to the low number of events. Furthermore, no data are available on the circulating virus variant (even if, considering the period in study, it is improbable that the enrolled were affected by delta variant); thus, the VE values should be interpreted considering a virus variant less contagious than the delta strain. Moreover, the Bari Policlinico health personnel were screened every 14 days for SARS-CoV-2 infection and so it is not impossible that some asymptomatic infection was not detected, even if improbable. Finally, the immune status (es. immunodepression, chemotherapy treatment, etc.) of enrolled subjects was not available. Further studies are needed to determine the VE of the BNT162b2 mRNA vaccine in different populations and over an even longer follow-up period.

Our data are consistent with the very high effectiveness of the BNT162b2 mRNA vaccine in the prevention of both SARS-CoV-2 infection and COVID-19 disease. Indeed, a 2021 study [18] evaluated the efficacy of the vaccine on antibody production and its side effects in health personnel with and without prior SARS-CoV-2 infection and in a group of unvaccinated individuals with prior COVID-19; the authors reported the production of 100% neutralizing antibodies in both groups after the second dose and well-tolerated adverse effects. On the other hand, Ioannu P et al. [19] reported a potentially reduced efficacy of BNT162b2 in preventing the transmission of variant B.1.1.7. These results may encourage vaccination among still-reluctant HCWs. Moreover, by preventing the infection of HCWs and limiting circulation of the virus in the nosocomial setting, especially among high-risk patients, vaccination is both a form of PPE and an ethical obligation to guarantee the safety of others. Vaccination hesitancy among Italian HCWs has thus far been tolerated and vaccination has yet to become mandatory. However, given the duration and seriousness of the pandemic and the emergence of more aggressive variants of the virus, this policy warrants very serious reconsideration. Indeed, on 31 March 2021, the Italian government made COVID-19 vaccination semi-compulsory, under penalty of salary suspension, but the practical effects of this regulation are not yet known.

A serious decrease in VE could require periodic re-vaccination, especially among HCWs. Indeed, in the UK, the Department of Health and Social Care recently announced the rollout of a COVID-19 booster vaccine in early autumn to protect the most vulnerable ahead of winter, when outbreaks of SARS-CoV-2 infections have been highest [20].

In conclusion, our study provides further evidence of the effectiveness and safety of the BNT162b2 mRNA vaccine, thus supporting its use as an essential weapon to end the COVID-19 pandemic. Nonetheless, in Italy and elsewhere, vaccination strategies must be strengthened, as too many HCWs and millions of people in the general population have yet to be vaccinated. Considering that the pandemic is far from over, stricter measures should be approved, including the mandatory vaccination of HCWs and stronger incentives within the general population.

## Figures and Tables

**Table 1:** Characteristics of the two study groups (vaccinated and unvaccinated HCWs) at baseline.

Variable.	Unvaccinated (*n* = 787)	Vaccinated (*n* = 5351)	Total (*n* = 6136)	*p*-Value
Age (Years); Mean ± SD (Range)	43.1 ± 12.8 (21–70)	44.9 ± 12.7 (22–70)	44.7 ± 12.8 (21–70)	<0.001
Sex;*n* (%)				
Female Male	489 (62.3) 289 (37.7)	3181 (59.5) 2170 (40.5)	3670 (59.8) 2466 (40.2)	0.129
Professional category; *n* (%)				
Physician Nurse Auxiliary staff Other HCWs	159 (20.3) 275 (35.0) 204 (26.0) 147 (18.7)	1.898 (35.5) 1.647 (30.8) 888 (16.6) 918 (17.1)	2.057 (33.5) 1.922 (31.3) 1.092 (17.8) 1.065 (17.4)	<0.0001

HCWs= healthcare workers.

**Table 2:** Characteristics of recorded symptoms, per group (vaccinated and unvaccinated HCWs).

Variable	Unvaccinated (*n* = 787)	Vaccinated (*n* = 5.351)	Total (*n* = 6.136)	OR (95%CI)	*p*-Value
Fever/hyperpyrexia; *n* (%)	66 (8.41)	6 (0.11)	72 (1.17)	81.5 (35.3–230.7)	<0.0001
Cough; *n* (%)	56 (7.13)	7 (0.13)	63 (1.03)	58.5 (26.4–152.5)	<0.0001
Dyspnea; *n* (%)	27 (3.44)	1 (0.02)	28 (0.46)	190.1 (31.2–7781.6)	<0.0001
Pharyngodynia; *n* (%)	21 (2.68)	6 (0.11)	27 (0.44)	24.4 (9.5–74.1)	<0.0001
Headache; *n* (%)	52 (6.62)	7 (0.13)	59 (0.96)	54.0 (24.3–141.3)	<0.0001
Ageusia/anosmia; *n* (%)	61 (7.77)	11 (0.21)	72 (1.17)	40.8 (21.2–86.4)	<0.0001

**Table 3:** Estimated vaccine effectiveness values for documented infection and symptomatic disease.

Period	Documented Infection	Symptomatic Disease
14–34 days after the first dose	97.7% (95.4–99.0%)	99.2% (96.4–99.8%)
14–41 days after the second dose	94.8% (87.0–97.8%)	97.2% (90.3–99.2%)
42–69 days after the second dose	83.0% (65.0–92.0%)	85.0% (63.0–94.2%)
>69 days after the second dose	81.0% (42.0–94.0%)	88.0% (42.0–97.6%)

## Data Availability

Data available on request due to restrictions, e.g., privacy or ethical.
